# Altered interpersonal distance regulation in autism spectrum disorder

**DOI:** 10.1192/j.eurpsy.2022.1892

**Published:** 2022-09-01

**Authors:** K. Farkas, O. Pesthy, A. Guttengéber, S. Weigl, E. Komoróczy, B. Szuromi, J. Réthelyi, D. Németh

**Affiliations:** 1 Semmelweis University, Department Of Psychiatry And Psychotherapy, Budapest, Hungary; 2 ELTE Eötvös Loránd University, Institute Of Psychology, Budapest, Hungary; 3 Université de Lyon, Lyon Neuroscience Research Center (crnl), Lyon, France

**Keywords:** personal space, Autism Spectrum Disorder, interpersonal distance, heart rate variability

## Abstract

**Introduction:**

Interpersonal distance regulation is an essential element of social communication. Its impairment in autism spectrum disorder (ASD) is widely acknowledged among practitioners, but only a handful of studies reported empirical research. However, these studies did not measure the alterations of vegetative functions related to interpersonal distance.

**Objectives:**

We introduced a new experimental design to systematically measure interpersonal distance along with heart rate variability (HRV) in adults with ASD and tested the modulatory effect of intentionality, eye contact, moving activity, and attribution.

**Methods:**

Twenty-two adults diagnosed with ASD and 21 matched neurotypical controls participated in our study from 2019 October to 2020 February. Our new experimental design combined the modified version of the stop distance paradigm with HRV measurement controlling for eye contact between the experimenter and the participant to measure interpersonal distance in incidental and intentional conditions.

**Results:**

Our results showed greater preferred distance in ASD in the intentional (*W*=103, *p*=0.002) but not in the incidental condition. These results were altered with eye contact and the participant’s role (active vs. passive) in the stop distance task (*F*(1,41)=6.150, *p*=0.017). Moreover, we found lower baseline HRV (*t* =-2.060, *p*=0.023) and reduced HRV reactivity in ASD; however, these vegetative measurements could not predict preferred interpersonal distance.

**Conclusions:**

Our study highlights the importance of interpersonal distance regulation in ASD and the need for comprehensive experimental designs to grasp the complexity and underlying factors of distance regulation in typical and atypical populations.

**Disclosure:**

No significant relationships.

